# Prognostic value of prelymphodepletion absolute lymphocyte counts in relapsed/refractory diffuse large B-cell lymphoma patients treated with chimeric antigen receptor T cells

**DOI:** 10.3389/fimmu.2023.1155216

**Published:** 2023-05-02

**Authors:** Yanyan Lu, Hong Zhu, Yang Liu, Ying Wang, Yinxiang Sun, Hai Cheng, Zhiling Yan, Jiang Cao, Wei Sang, Feng Zhu, Depeng Li, Haiying Sun, Junnian Zheng, Kailin Xu, Zhenyu Li

**Affiliations:** ^1^ Blood Diseases Institute, Xuzhou Medical University, Xuzhou, Jiangsu, China; ^2^ Department of Hematology, The Affiliated Hospital of Xuzhou Medical University, Xuzhou, Jiangsu, China; ^3^ Key Laboratory of Bone Marrow Stem Cell, Xuzhou, Jiangsu, China; ^4^ Cancer Institute, Xuzhou Medical University, Xuzhou, Jiangsu, China; ^5^ Jiangsu Center for the Collaboration and Innovation of Cancer Biotherapy, Cancer Institute, Xuzhou, Medical University, Xuzhou, Jiangsu, China; ^6^ Center of Clinical Oncology, The Affiliated Hospital of Xuzhou Medical University, Xuzhou, Jiangsu, China

**Keywords:** chimeric antigen receptor T cell, diffuse large B-cell lymphoma, prelymphodepletion absolute, prognostic value, survival time

## Abstract

**Introduction:**

Chimeric antigen receptor (CAR) T cell therapy has achieved unprecedented efficacy recently. However, the factors related to responses and durable remission are elusive. This study was to investigate the impact of pre-lymphodepletion (pre-LD) absolute lymphocyte count (ALC) on CAR T cell therapy outcomes.

**Methods:**

We conducted a retrospective study of 84 patients with relapsed/refractory diffuse large B-cell lymphoma (R/R DLBCL) who underwent CAR T cell treatment at the Affiliated Hospital of Xuzhou Medical University between March 1,2016 and December 31, 2021. The enrolled patients were divided into high group and low group according to the optimal cutoff value of pre-LD ALC. The Kaplan-Meier analyses was used to calculate survival curves. The Cox proportional hazards model was used for univariate and multivariate analysis to assess the prognostic factors.

**Results:**

The ROC showed that the optimal cutoff value of pre-LD ALC was 1.05 x 10^9^/L. The overall response (defined as partial response or complete response) rate was significantly higher in patients with a high pre-LD ALC (75% versus 52.08%; P=0.032). Patients with a low pre-LD ALC had significantly inferior overall survival (OS) and progression-free survival (PFS) compared with those having a high pre-LD ALC (median OS, 9.6 months versus 45.17 months [P=0.008]; median PFS, 4.07 months versus 45.17 months [P= 0.030]). Meanwhile, low pre-LD ALC is an independent risk factor for PFS and OS.

**Discussion:**

The data suggested that pre-LD ALC may serve as a helpful indicator to predict the outcomes of CAR T cell therapy in patients with R/R DLBCL.

## Introduction

With a prevalence of about 24% non-Hodgkin’s lymphomas (NHL), diffuse large B-cell lymphoma (DLBCL) is a common lymphoma group whose prevalence increases with age (1). DLBCL has achieved a high cure rate in combination therapy with Rituximab-based treatment. However, there are approximately 10%-15% of patients no response, and 20%-30% of patients relapse (2–4). CAR T cell therapy has achieved significant efficacy in clinical trials targeting R/R B-NHL (5). At present, the two FDA (Food and Drug Administration)-approved anti-CD19 CAR products axicabtagene ciloleucel (6) and tisagenle cleucel (7), have achieved 52%-82% overall response rates (ORR) and 40%-54% complete remission rate (CRR) in DLBCL.

Studies have confirmed that the higher peak of blood CAR T cells expansion *in vivo* is a crucial indicator of therapeutic efficacy and associated with the persistence of responses. The persistence and function of CAR T cells are also correlated with the immunophenotype of T cell subsets (8, 9). Jacobson et al. (10) found that high c-reactive protein (CRP) levels on day 0 after CAR T cells infusion and larger tumor volume (>5cm) were associated with poor prognosis. However, no association was found between International Prognostic index (IPI), double or triple hit lymphoma, number of previous treatment lines, bridging therapy, cytokine release syndrome (CRS) or neurotoxicity, use of tocilizumab or steroid and efficacy in this study related to axicabtagene ciloleucel. Regrettably, the existing predictors are too less and not sufficiently convenient and economical. This current compels us to find more convenient indicators to identify which patients can most benefit from CAR T cell treatment.

The immune status is closely related to the outcome and prognosis of the disease. ALC, which represents the immune status of patients, is a prognostic factor of a variety of hematologic malignancies (11–13). Previous findings have demonstrated that lymphopenia is a poor prognostic indicator for solid malignancies (14) as well as hematological malignancies such as lymphoma (15) and acute myeloid leukemia (AML) (16). A meta-analysis of six studies involving 1206 patients with DLBCL confirmed the adverse effect of low ALC in patients who received CHOP combined with or without rituximab (17). Yet, whether pre-lymphodepletion (pre-LD) ALC could be a predictor in R/R DLBCL patients receiving CAR T cell therapy remains unclear. In this study, we assessed the impact of pre-LD ALC on the prognosis of 84 patients with R/R DLBCL treated with CAR T cells.

## Methods

### Patient selection

This is a retrospective study. 84 patients with R/R DLBCL were enrolled in the Affiliated Hospital of Xuzhou Medical University between March 1,2016 and December 31, 2021 (ChiCTR 2100049709). All enrolled patients met the 2016 World Health Organization (WHO) diagnostic criteria of the lymphoid neoplasms (18). Patients who were pregnant, nursing, or likely to become pregnant and those with mental or psychological illnesses, severe allergies, a history of severe allergies were excluded. Clinical data involved age, gender, Ann Arbor stage, IPI, previous therapies, complete blood count and lactate dehydrogenase (LDH) before lymphocyte depletion. The clinical trial has been approved by the Institutional Review Committee of the Affiliated Hospital of Xuzhou Medical University in accordance with the provisions of the Helsinki Declaration. All enrolled patients or their family members signed an informed consent form prior to the study.

### CAR T cell manufacture and infusion

Peripheral blood mononuclear cells were collected by COMTEC hemocytometer (Fresenius Kabi [China] Investment Co, LTD.) for anti-CD19, anti-CD20 and anti-CD22 CAR T cells generation. Before CAR T cells infusion, 82 patients received lymphodepletion chemotherapy consisting of fludarabine (3 daily doses of 30mg/m2 on days -5 to -3) and cyclophosphamide (1 daily dose of 750 mg/m2 on day -5). Anti-CD19 CAR T cells alone or in combined with anti-CD22 or anti-CD20 CAR T cells were infused into the enrolled patients on day 0.

### Evaluation criteria for efficacy and adverse reactions

All patients were evaluated according to the 2014 Lugano efficacy evaluation criteria (19). Responses were assessed as complete response (CR), partial response (PR), stable disease (SD), progressive disease (PD). The overall response rate (ORR) was defined as ORR=CR+PR rates. Blood pressure, respiration, heart rate and other vital signs were detected during cell infusion. The main adverse reactions were CRS, which graded and managed according to the recommendations of Lee et al. (20).

### Followed-up

The follow-up included an outpatient follow-up, a telephone follow-up and access to hospital records. The follow-up period was until December 31, 2021. PFS was defined as the time from CAR T cell infusion to disease progression, relapse or death. OS was determined as the time from CAR T cell infusion to death from any cause, loss of visit or final follow-up.

### Statistical analysis

All data were analyzed using SPSS (version 25.0, SPSS Inc. Chicago, IL, USA). Categorical variables were analyzed using Pearson chi-square test, which is expressed as percentage. Two independent samples T test was used for two normally distributed continuous independent variables expressed as mean ± standard deviation while Mann-Whitney rank sum test was used for that not normally distributed variables expressed as the median and quartile. Kaplan-Meier analyses were used to describe survival curve. Cox proportional hazards model was used for univariate and multivariate analysis of PFS and OS. All tests were 2-sided, and a P value <.05 indicated a significant difference.

## Results

### Patient characteristics

A total of 84 patients with R/R DLBCL were enrolled, and the basic characteristics are shown in **Table 1**. Fifty-five patients were male and twenty-nine patients were female. The median age was 51 years (range, 21 to 72 years) and 50% patients were over 60 years old. The median time from diagnosis to enrollment was 15.27 months. Three patients had undergone previous autologous hematopoietic stem cell transplantation (ASCT) and twenty-five had previous immunotherapy. There were fifty patients with elevated LDH. Sixty-three patients (75%) were in stage III or IV status and twenty-three patients (27.38%) had B symptoms according to the Ann Arbor clinical stage. Thirty-six patients (42.86%) had ECOG score of 2, and 30 patients (35.71%) had IPI score in medium-high risk to high-risk group. Thirty-one patients received anti-CD19 and anti-CD22 CAR T cell infusion; seventeen patients received anti-CD19 and anti-CD20 CAR T cell infusion; and thirty-six patients received anti-CD19 CAR T cell infusion alone.

**Table 1:** Baseline characteristics of 84 patients with R/R DLBCL.

Baseline characteristic		Number	%
Gender	male	55	65.48
	female	29	34.52
Ann Arbor stage	I	12	14.3
	II	9	10.70
	III	21	25.00
	IV	42	50.00
B symptoms	absence	61	72.62
	presence	23	27.38
CNS infiltration	No	66	78.57
	Yes	18	21.43
Bone marrow infiltration	No	58	69.05
	Yes	26	30.95
Prior immunotherapy	No	59	70.24%
	Yes	25	29.76%
Prior ASCT	No	81	96.43
	Yes	3	3.57
Use of Corticosteroids	No	55	65.48
	Yes	29	34.52
Use of Tocilizumab	No	76	90.48
	Yes	8	9.52
Followed immunotherapy	No	59	70.20
	Yes	25	29.80
ECOG	0	1	1.19
	1	47	55.95
	2	36	42.86
IPI	1	22	26.19
	2	32	38.10
	3	23	27.38
	4	7	8.33
CRS	0	21	25.00
	1	31	36.91
	2	23	27.38
	3	5	5.95
	4	4	4.76
Clinical response	PD+SD	32	38.10
	PR+CR	52	61.90

CNS, central nervous system.

ASCT, autologous hematopoietic stem cell transplantation.

IPI, International Prognostic index.

CRS, cytokine release syndrome.

### Factors associated with pre-LD ALC

**Figure 1:**
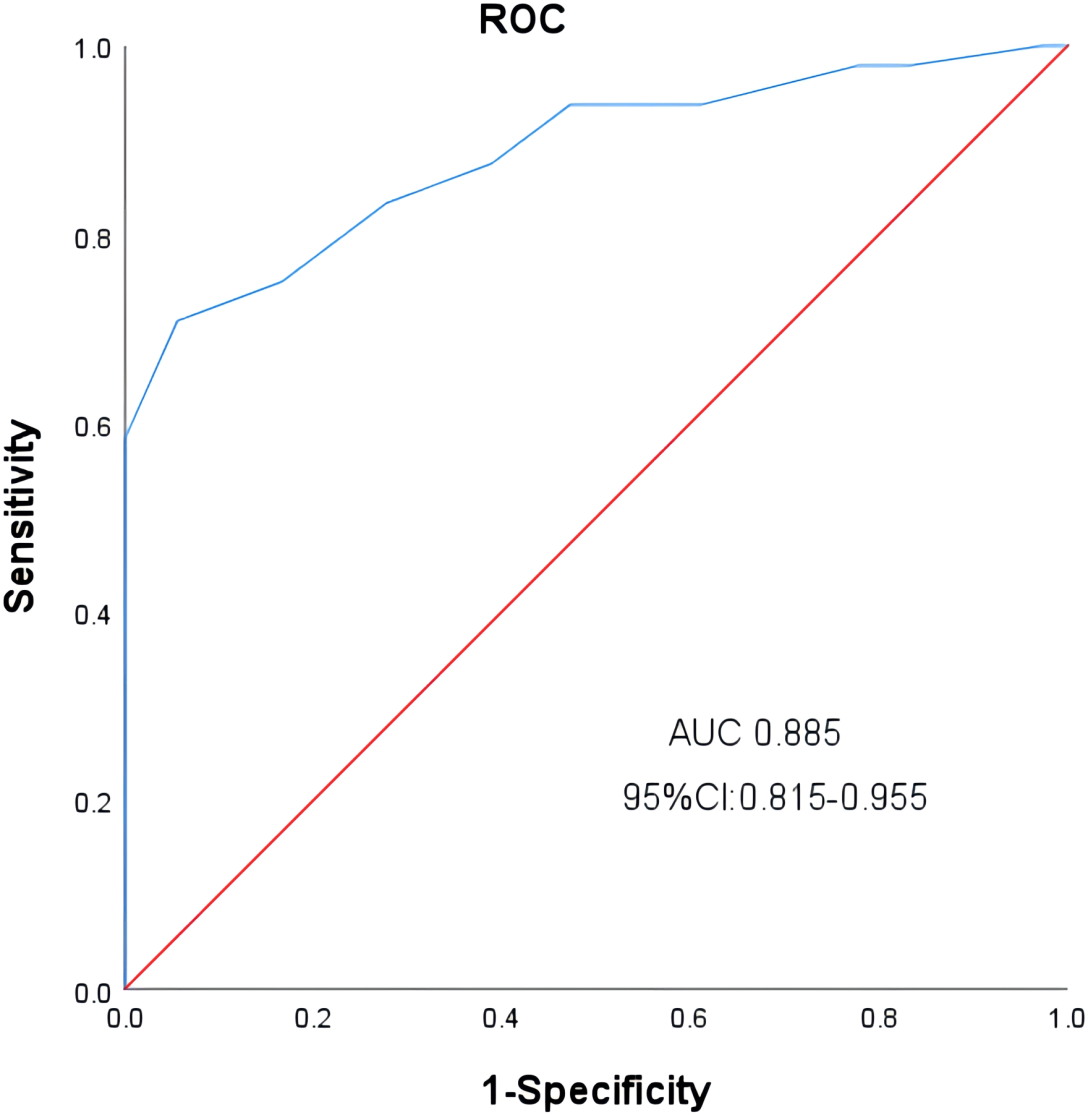
ROC analysis of Pre-LD ALC.

We obtained the optimal cutoff value of pre-LD ALC by receiver operating characteristic curve. The cutoff value was 1.05 x 10^9^/L (sensitivity, 0.708; specificity, 0.944; area under the curve, 0.885; 95% confidence interval [CI], 0.815-0.955) (**Figure 1**). According to the optimal cut-off value of ALC, 48 patients were included in the low ALC group (<1.05 x 10^9^/L), and 36 patients were included in the high ALC group (≥1.05 x 10^9^/L). Compared to the high pre-LD ALC group, the low group had more patients with higher IPI score, worse clinical response, higher LDH and neutrophil-to-lymphocyte ratio (NLR), lower lymphocyte-to-monocyte ratio (LMR). There were statistically significant differences on IPI (p = 0.004), clinical response (p = 0.032), LDH (p = 0.039), NLR (p = 0.002), LMR (p<0.001). On the contrary, the two groups’ age, gender, ECOG, Ann Arbor stage, B symptoms, use of corticosteroids or tocilizumab etc. had no significant difference (**Table 2**). Particularly worth mentioning is that we did not find the relationship between pre-LD ALC and CRS (**Table 2**).

**Table 2:** Factors associated with pre-LD ALC.

	Low pre-LD ALC(n=48)	High pre-LD ALC(n=36)	*P*
Gender, n (%)			0.098
Male Female	35 (72.92)	20 (55.56)	
	13 (27.08)	16 (44.44)	
Ann Arbor stage, n (%)			0.300
1	4 (8.33)	8 (22.22)	
2	5 (10.42)	4 (11.11)	
3	14 (29.17)	7 (19.44)	
4	25 (52.08)	17 (47.22)	
B symptoms, n (%)	12 (25.00)	11 (30.56)	0.572
CNS infiltration, n (%)	8 (16.67)	10 (27.78)	0.219
Bone marrow infiltration, n (%)	17 (35.42)	9 (25.00)	0.307
Prior immunotherapy, n (%)	13(27.08)	12(33.33)	0.535
Prior ASCT, n (%)	1 (2.08)	2 (5.56)	0.396
Use of steroids, n (%)	15 (31.25)	14(38.89)	0.466
Use of Tocilizumab, n (%)	4 (8.33)	4 (11.11)	0.668
Followed immunotherapy, n (%)	16 (33.33)	9 (25.00)	0.408
ECOG, n (%)			0.081
0	1 (2.08)	0 (0.00)	
1	22 (45.83)	25 (69.44)	
2	25(52.08)	11 (30.56)	
IPI, n (%)			0.004
1	9 (18.75)	13 (36.11)	
2	14 (29.17)	18 (50.00)	
3	19 (39.58)	4 (11.11)	
4	6 (12.50)	1 (2.78)	
CRS, n (%)			0.979
0	11 (22.92)	10 (27.78)	
1	18 (37.50)	13 (36.11)	
2	14 (29.17)	9 (25.00)	
3	3 (6.25)	2 (5.56)	
4	2 (4.17)	2 (5.56)	
Clinical response, n (%)			0.032
PD+SD	23(47.92)	9(25.00)	
PR+CR	25(52.08)	27(75.00)	
Previous treatment lines, median (quartile)	3(2; 6)	4(3; 5)	0.511
Pre-LD LDH, U/L, median (quartile)	340.00 (218.50; 604.25)	257.00 (185.00; 381.75)	0.039
Pre-LD ANC, x 10^9^/L, median (quartile)	2.87 (1.54; 4.32)	3.59 (2.27; 4.89)	0.072
Pre-LD AMC, x 10^9^/L, mean ± standard	0.50 ± 0.25	0.41 ± 0.18	0.071
Pre-LD NLR, median (quartile)	3.74 (2.52; 6.82)	2.45(1.46; 3.73)	0.002
Pre-LD LMR, median (quartile)	1.35 (0.92; 2.31)	3.56(2.85; 5.52)	<0.001
CRP peak, mg/L, median (quartile)	71.60 (18.90; 115.90)	47.55 (10.70; 151.68)	0.591
FER peak, mg/ml, median (quartile)	959.40 (550.95; 1969.00)	682.45 (275.72; 1929.50)	0.169
IL-6 peak, pg/ml, median (quartile)	39.47 (22.76; 114.33)	39.12 (13.81; 98.79)	0.381
Age, yr, median (quartile)	59.50 (40.50; 64.25)	61.00 (42.00; 66.00)	0.415
Time from diagnosis to enrollment, m, median (quartile)	14.79 (9.47; 28.86)	16.52 (11.75; 24.36)	0.889

CNS, central nervous system.

ASCT, autologous hematopoietic stem cell transplantation.

IPI, International Prognostic index.

CRS, cytokine release syndrome.

CR, complete response.

PR, partial response.

SD, stable disease.

PD, progressive disease.

LDH, lactate dehydrogenase.

ANC, absolute neutrophil count.

AMC, absolute monocyte count.

NLR, neutrophil-to-lymphocyte ratio.

LMR, lymphocyte-to-monocyte ratio.

CRP, c-reactive protein.

FER, ferritin.

IL-6, interleukin-6.

### Survival analysis based on pre-LD ALC

**Figure 2:**
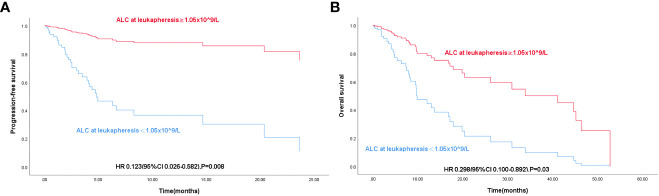
**(A)** K-M curve of progression-free survival in 84 patients with R/R DLBCL according to Pre-LD ALC grouping. **(B)** K-M curve of overall survival in 84 patients with R/R DLBCL according to Pre-LD ALC grouping.

In low pre-LD ALC group, the median PFS was 4.07 and the median OS was 9.6; In high pre-LD ALC group, the median PFS was 45.17 and the median OS was also 45.17. The results showed that OS (P< 0.001) and PFS (P< 0.001) in the low group were significantly shorter than those in the high group (P< 0.001) (**Figures 2A, B**) according to Kaplan-Meier survival curve.

### Univariate and multivariate analysis of PFS and OS

By cox univariate regression analysis, we found that Ann Arbor stage, ECOG score and IPI score, increased LDH, ALC < 1.05 x 10^9^/L, increased NLR and decreased LMR were risk factors affecting OS and PFS in DLBCL patients (P < 0.05) (**Tables 3**, **4**). In addition, PFS was also affected by increased monocyte (AMC) (P < 0.05) (**Table 3**). The significant variables in univariate regression analysis were included in the multivariate analysis model, and the results showed that elevated LDH (HR = 1.002, 95%CI: 1.001-1.003, P < 0.001) and ALC < 1.05 x 10^9^/L (HR = 0.299, 95%CI: 0.100-0.893, P = 0.030) were an independent risk factor for OS in patients with DLBCL (**Table 4**). IPI score (HR = 1.614, 95%CI: 1.011-2.576, P = 0.045), elevated LDH (HR = 1.002, 95%CI: 1.001-1.002, P< 0.001) and ALC < 1.05 x 10^9^/L (HR = 0.129, 95%CI: 0.027-0. 605, P = 0.009) were independent risk factors for PFS in patients with DLBCL (**Table 3**).

**Table 3:** Univariate and multivariate analysis of PFS.

	Univariate analysis			Multivariate analysis		
	P	95%CI		P	95%CI	
Age	0.470	0.969	1.015		
Gender	0.121	0.244	1.178		
Time from diagnosis to enrollment	0.242	0.977	1.006		
Ann Arbor stage	0.025	1.056	2.216	0.785	0.681	1.662
B symptoms	0.187	0.790	3.346		
CNS infiltration	0.906	0.459	2.404		
Bone marrow infiltration	0.784	0.452	1.820		
Prior immunotherapy	0.148	0.829	3.472		
Prior ASCT	0.987	0.134	7.213		
Use of Corticosteroids	0.243	0.761	2.935		
Use of Tocilizumab	0.058	0.965	8.149		
Followed immunotherapy	0.452	0.651	2.617		
ECOG	0.025	1.097	4.090	0.595	0.376	1.753
IPI	<0.001	1.563	3.493	0.045	1.011	2.576
CRS	0.224	0.891	1.633		
Previous treatment lines	0.066	0.954	4.454		
LDH	<0.001	1.001	1.003	<0.001	1.001	1.002
Pre-LD ALC	<0.001	0.018	0.305	0.009	0.027	0.605
Pre-LD ANC	0.227	0.932	1.344		
Pre-LD AMC	0.009	1.653	33.499	0.091	0.736	64.626
NLR	<0.001	1.062	1.162	0.208	0.975	1.125
LMR	0.029	0.646	0.977	0.163	0.947	1.383
CRP peak	0.074	1.000	1.007		
FER peak	0.952	1.000	1.000		
IL-6 peak	0.258	1.000	1.001		
Clinical response	0.096	0.280	1.110		

CNS, central nervous system.

ASCT, autologous hematopoietic stem cell transplantation.

IPI, International Prognostic index.

CRS, cytokine release syndrome.

LDH, lactate dehydrogenase.

ALC, absolute lymphocyte count.

ANC, absolute neutrophil count.

AMC, absolute monocyte count.

NLR, neutrophil-to-lymphocyte ratio.

LMR, lymphocyte-to-monocyte ratio.

CRP, c-reactive protein.

FER, ferritin.

IL-6, interleukin-6.

**Table 4:** Univariate and multivariate analysis of OS.

	Univariate analysis			Multivariate analysis		
	P	95%CI		P	95%CI	
Age	0.898	0.979	1.024		
Gender	0.393	0.392	1.445		
Time from diagnosis to enrollment	0.120	0.998	1.016		
Ann Arbor stage	0.031	1.035	2.091	0.441	0.77	1.82
B symptoms	0.729	0.537	2.430		
CNS infiltration	0.486	0.584	3.094			
Bone marrow infiltration	0.454	0.400	1.505		
Prior immunotherapy	0.088	0.235	1.105		
Prior ASCT	0.971	0.141	7.641		
Use of Corticosteroids	0.055	0.986	3.859		
Use of Tocilizumab	0.226	0.629	7.078		
Followed immunotherapy	0.054	0.988	3.845		
ECOG	0.091	0.917	3.259		
IPI	0.006	1.153	2.309	0.458	0.795	1.663
CRS	0.942	0.746	1.312		
Previous treatment lines	0.623	0.620	2.225		
LDH	<0.001	1.001	1.003	<0.001	1.001	1.003
Pre-LD ALC	<0.001	0.066	0.431	0.030	0.100	0.893
Pre-LD ANC	0.463	0.775	1.123		
Pre-LD AMC	0.116	0.779	9.680		
NLR	<0.001	1.069	1.197	0.159	0.976	1.157
LMR	0.002	0.559	0.877	0.855	0.798	1.205
CRP peak	0.056	1.000	1.008		
FER peak	0.052	1.000	1.000		
IL6 peak	0.453	1.000	1.001		
Clinical response	0.091	0.295	1.094		

## Discussion

DLBCL is a hematological malignancy with high heterogeneity. IPI, as the scoring system based on clinical and pathological features, is widely used to evaluate the prognosis of patients with DLBCL. However, it cannot really reflect the heterogeneity of cell morphology, immunophenotype and molecular biology in DLBCL patients, and the prognosis varies dramatically in patients with the same IPI score. Currently, finding new prognostic indicators with high accuracy and specificity is a hot spot in clinical diagnosis and treatment of DLBCL (21, 22).

It has been shown that the tumor microenvironment (TEM), which is composed of immune cells, stromal cells, and blood vessels, is crucial to the pathogenesis and clinical course of DLBCL. The tumor microenvironment can effectively identify tumors, and provide protection for tumor cells, promoting their growth and survival (23, 24). Also, it can help cancer cells escape host immune surveillance due to its interaction with lymphoma cells (25). Lymphocyte, an immune cell in TEM, has been shown to influence lymphoma patient survival. It plays a significant role in the immune surveillance in tumor patients and its depletion has been proved to be a key marker of host immunodeficiency (26). Studies (27–29) have shown that ALC can be used as an independent prognostic factor in leukemia, breast cancer, multiple myeloma, as well as other malignant tumors.

In this study, 57.0% of 84 patients with R/R DLBCL showed a decrease in ALC before lymphodepletion, possibly due to the toxic lytic effect of cytokines secreted by lymphoma cells on lymphocytes. We observed an ORR of 61.9%, which is similar to the known findings (7). High pre-LD ALC was associated with higher ORR and longer survival. Previous studies (30, 31)have shown that decreased ALC and B symptoms have been reported as poor prognostic indicators for NHL, which are independent of IPI and treatment regimen. We found PFS (P < 0.001) and OS (P < 0.001) in the high pre-LD ALC group were significantly longer than those in the low pre-LD ALC group, suggesting that pre-LD ALC is closely related to the prognosis of DLBCL patients and patients with low pre-LD ALC had a poor prognosis. In order to further explore the independent prognostic value of ALC, we then performed COX regression analysis. In univariate survival analysis, low LMR and pre-LD ALC < 1.05 x 10^9^/L were risk factors for OS and PFS. But low LMR lost its prognostic value in multivariate analysis. Only pre-LD ALC < 1.05 x 10^9^/L was an independent risk factor for OS and PFS in patients with DLBCL treated with CAR T cell. The reason may be that the immune response during chemotherapy is relatively weak in those with low lymphocytes, and the killing effect on tumor cells is small. NLR and LMR, as inflammatory biomarkers, represent the balance between the host immune system and TEM, and their changes will affect the normal immune response, immune prevention and other important functions (32, 33). Nevertheless, the synergistic effect of lymphocyte, monocyte and neutrophil may affect the predictive ability of LMR and NLR, which explains why their prognostic value were lost in the multifactorial analysis. In addition, we found that there were no statistically significant differences in Ann Arbor stage, age, B symptoms, ECOG, etc. between the two groups divided by pre-LD ALC of 1.05 x 10^9^/L. The reason may be that lymphocyte immunoregulation is impaired, thus affecting the immune suppression and immune killing of lymphoma cells.

Since its formal introduction in 1993, IPI has been widely used to evaluate risk stratification of DLBCL for nearly 30 years. According to five indicators including age, LDH, ECOG score, Ann Arbor stage, and number of extra nodal involvement, patients are divided into four risk groups: low-risk, intermediate-low risk, intermediate-high risk, and high-risk. The 5-year OS rates were 84.5%, 70.1%, 53.1% and 64.2%, respectively (34). However, in an analysis of the prognosis of patients in the R-CHOP regimen group, Ngo (35)concluded that there was no significant difference in the 5-year OS rates between the low-risk groups and the intermediate-low risk groups, and between the intermediate-high risk groups and the high-risk groups, suggesting that in the era of rituximab, IPI was not sufficient to distinguish DLBCL with different prognosis. Likewise, IPI was not sufficient to predict OS independently in our study. As for malignant neoplastic diseases, malignant proliferation of tumor cells requires a large amount of energy consumption causing an increased glycolytic response, which in turn causes an increase LDH synthesis in patients (36). Excessive LDH has long been shown to be associated with poor prognosis in DLBCL, and similarly, elevated LDH was an independent risk factor for OS and PFS in our research. Whether ALC can be combined with IPI to form a new prognostic scoring system also requires further research and external validation.

The most common adverse effects of CAR T cell therapy are CRS and neurotoxic reactions. CRS are usually associated with cytokine levels, tumor burden and number of CAR T cell infusion and expansions. We have not found a correlation between Pre LD ALC and CRS. Previous studies have reported that the incidence of CRS in R/R B-NHL treated with CAR T cell ranged from 37% to 93% (37). Also, the occurrence of CRS can indicate the response to CAR T cell therapy to some extent. Patients without CRS often have the worse efficacy (38). In this study, 75% patients occurred CRS, mainly grade 1, which may be related to the fact that we reduced the tumor burden of patients as far as possible before infusion, such as FC regimen pretreatment and controlling the number of cell infusion. Corticosteroids attenuate toxicity by inhibiting CAR T cell proliferation and inflammatory cytokine production, but their effect on CAR T cell expansion and durable response remains unknown (39). In ZUMA-1 study (6), tocilizumab and corticosteroids were used primarily for grade ≥3 CRS and neurotoxic reactions, and the use of them did not seem to influence ORR or sustained response. In our univariate and multivariate survival analyses, no effect of tocilizumab or corticosteroid use on survival was found (P > 0.05). Nonetheless, the dose and duration of corticosteroid and tocilizumab after CAR T cell therapy remain clinically unanswered challenge.

This study has some limitations, such as single-center study, small sample size, short follow-up time. Therefore, studies with more samples are needed to confirm the potential prognostic value of pre-LD ALC in CAR T cell therapy. At the same time, external verification may improve our confidence in drawing solid conclusions.

In conclusion, our findings indicate that low pre-LD ALC is associated with inferior outcomes in R/R DLBCL patients treated with CAR T cells. Pre-LD ALC can serve as an independent prognostic indicator for these patients.

## Data availability statement

The original contributions presented in the study are included in the article/**Supplementary Material**. Further inquiries can be directed to the corresponding authors.

## Ethics statement

The studies involving human participants were reviewed and approved by the ethics committee of the Affiliated Hospital of Xuzhou Medical University. The patients/participants provided their written informed consent to participate in this study. Written informed consent was obtained from the individual(s) for the publication of any potentially identifiable images or data included in this article.

## Author contributions

YYL, HZ, YL, and ZL proposed the project. ZL, KX, and JZ supervised the clinical study. YW, YS, HC, ZY, JC, WS, FZ, DL, and HS were responsible for patient recruitment. YYL and HZ collected and analyzed data, wrote the manuscript. YL, YS, YW, HC, ZY, JZ, KX, and ZL helped revise the manuscript. All authors discussed the data and the analytic methods and contributed to the final draft. YYL, HZ, and YL contributed equally to this work and should be considered co-first authors. All authors contributed to the article and approved the submitted version.
